# Cortical Blindness and Thrombotic Microangiopathy Following a Hemotoxic Snake Envenomation: An Unusual Presentation

**DOI:** 10.7759/cureus.43109

**Published:** 2023-08-08

**Authors:** Anitha Ramkumar, Murthy TVSP, Rajkumar Elanjeran, Y Vishnu Chaitanya, Kari Harika

**Affiliations:** 1 Department of Emergency Medicine, Aarupadai Veedu Medical College, Vinyaka Mission Research Foundation (Deemed University), Puducherry, IND; 2 Department of Anaesthesiology, GSL Medical College, Rajahmundry, IND; 3 Department of Emergency Medicine, GSL Medical College, Rajahmundy, IND; 4 Department of Emergency Medicine, GSL Medical College, Rajahmundry, IND

**Keywords:** snake venom, venom induced consumptive coagulopathy, hemotoxic, thrombotic microangiopathy, snake envenomation, cortical blindness

## Abstract

Snake envenomation leads to significant morbidity and mortality with local, hematological, renal, and neurological complications. Hemotoxic envenomation activates a hemorrhagic cascade, leading to many manifestations ranging from skin bleeds to major organ bleeds and fatal intracranial hemorrhage. Although rare, ischemic manifestations are possible following a hemotoxic envenomation, and they may present as cortical blindness, an unusual ocular symptom. Snake envenomation is also known to cause multifactorial acute kidney injury (AKI), precipitated by hemodynamic disturbances secondary to rhabdomyolysis, hemoglobinuria, direct tubular toxicity, and thrombotic microangiopathy. Thrombotic microangiopathy (TMA) is often overlooked in snake bites, as the hematological manifestations are often conveniently attributed to venom-induced consumptive coagulopathy (VICC). The distinct clinical entity of thrombotic microangiopathy should factor into one’s differential diagnosis in patients presenting with microangiopathic hemolytic anemia, thrombocytopenia, and acute kidney injury following a snake bite. We report a patient who developed cortical blindness and thrombotic microangiopathy following snake envenomation, which are uncommon sequelae.

## Introduction

Snake envenomation is a disease of the poor and marginalized, with significant morbidity and mortality, especially in the tropics [1]. Venomous snakebites result in diverse multisystem manifestations of neurologic or hemostatic dysfunction, depending on the envenomation type. This paper highlights two pathologies of particular interest that are often overlooked: cortical blindness and thrombotic microangiopathy (TMA). Common ocular manifestations are ptosis and external ophthalmoplegia, which are often the presenting signs of a neuroparalytic snake bite [2]. Although bilateral visual loss as an ocular presentation following snake envenomation is not uncommon, cortical blindness following a hemotoxic snake envenomation is rarely reported [3]. It is also important to point out that thrombotic microangiopathy following a hemotoxic snake envenomation often mistakenly attributes the hematologic derangement to disseminated intravascular coagulation (DIC) following venom-induced consumptive coagulopathy (VICC) [4]. Our patient presented with bilateral vision loss due to multiple occipital infarcts, and clinicopathologic evaluation was suggestive of thrombotic microangiopathy as evidenced by microangiopathic hemolytic anemia, renal failure, and thrombocytopenia. Apart from direct and established effects due to coagulopathy, the combination of toxic vasculitis, thrombotic microangiopathies, widespread vasospasm, and endothelial damage can cause tissue ischemia, leading to clinical presentation [3].

## Case presentation

A 50-year-old female presented to the emergency department with complaints of bilateral loss of vision and anuria. The patient allegedly had a history of a bite of unknown nature over the dorsum of her foot while sleeping on the ground at her residence approximately eight hours before the onset of symptoms. On clinical examination, two distinct fang marks were noted over the dorsum of the foot, with minimal induration and edema. The systemic examination was unremarkable, and she was hemodynamically stable. Fundoscopy and cranial nerve examination of both eyes revealed a normal cornea, lens, vitreous humor, retina, and optic disc, no perception of light, and normal pupillary reflexes. The patient did not have any bleeding manifestations on presentation.

The patient was subjected to hematological investigations, which were suggestive of possible hemotoxic snake envenomation, as evidenced by a positive 20-minute whole blood clotting time, a deranged coagulation profile, elevated D-dimer, low fibrinogen, elevated creatinine phosphokinase, and altered kidney function tests suggestive of acute kidney injury (Table 1).

**Table 1:** Investigation trends in the patient following the snake envenomation Positive 20-minute WBCT, deranged coagulation profile, elevated D-dimer, and hypofibrinogenemia suggestive of venom-induced consumptive coagulopathy (VICC); † Deranged renal function tests suggest an acute kidney injury; ǂ Microangiopathic hemolytic anemia as evidenced by anemia, indirect hyperbilirubinemia, increased LDH, thrombocytopenia, and deranged renal function tests suggestive of thrombotic microangiopathy Hb: hemoglobin; WBC: white blood cell count; WBCT: whole blood clotting time; SGOT: serum glutamic oxaloacetic transaminase; SGPT: serum glutamate pyruvate transaminase; PT: prothrombin time; INR: international normalized ratio; LDH: lactate dehydrogenase; NA: not applicable.

Investigations	Day 1	Day 2	Day 3	Day 4	Day 5	Day 6	Day 8	Day 10	Day 12
Hb (g/dl)	13	12	10	8ǂ	8.9	10.9	11	12	10.9
WBC (cells/mm^3^)	15600	11600	11800	10600	8600	9200	9600	10300	9700
Platelet count (cells/mm^3^)	1.25 Lakh	1.3 Lakh	1 lakh	80,000ǂ	1.1 Lakh	1.3 Lakh	1.46 Lakh	1.5 Lakh	3.43 Lakh
20-minute WBCT	Positive	Negative	NA	NA	NA	NA	NA	NA	NA
Total bilirubin (mg/dl)	1.3	1.6	1.4	2.8ǂ	1.8	1.2	1.2	0.8	0.8
Direct bilirubin (mg/dl)	0.4	0.5	0.4	0.4	0.4	0.6	0.6	0.4	0.4
SGOT (IU/L)	158	68	32	28	32	34	34	34	36
SGPT(IU/L)	46	40	40	30	36	38	44	40	42
Blood urea (mg/dl)	73 †	86 †	78 †	91 †ǂ	125 †	106 †	88 †	56 †	48 †
Serum creatinine (mg/dl)	2.2 †	3.3 †	3.6 †	4.3 †ǂ	6.6 †	7.4 †	5.6 †	3.3 †	3.0 †
PT Test/control (seconds)	37.8/10.5	14/10.2	12/10.1	12/10.2	13/10.2	12/10.1	11/10.6	12/10.2	13/10.2
INR	3.6	1.37	1.18	1.1	1.2	1.1	1.03	1.17	1.2
D-dimer (ng/ml)	>10000	5463	2800	480	462	438	400	428	446
LDH	Not done	Not done	Not done	3450ǂ	2860	1200	480	280	240
Creatine phosphokinase (IU/L)	1440	860	180	Not done	Not done	Not done	Not done	Not done	Not done
Serum fibrinogen(ng/ml)	120	140	280	360	320	380	Not done	Not done	Not done

**Figure 1:**
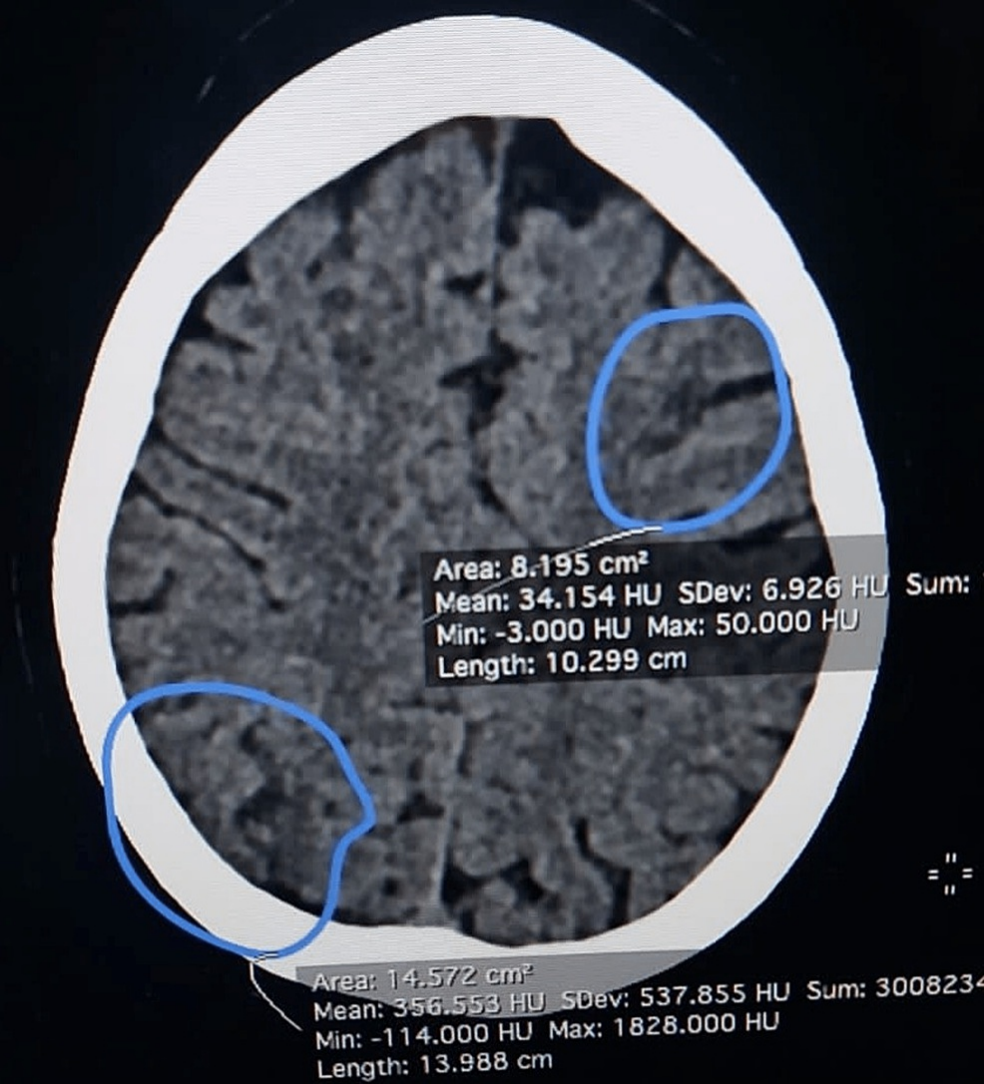
A CT brain image shows multiple acute infarcts in the right posterior parietal sulcus, right parietal subcortical white matter, left supraventricular level, and cerebellar hemisphere.

A CT scan of the brain revealed multiple acute infarcts in the right posterior parietal sulcus, right parietal subcortical white matter, left supraventricular level, frontal lobe subcortical white matter, and left cerebellar hemisphere (Figure 1).

The clinical syndrome and investigations were presumably attributed to a hemotoxic Viperidae snake envenomation. The patient was admitted to our intensive care unit, and 10 vials of polyvalent anti-snake venom (ASV) were administered (snake venom antiserum I.P., manufactured by Premium Serums and Vaccines Pvt. Ltd. (PSVPL), Junnar, India) after premedication with antihistamines and steroids. Additionally, 10 vials more of ASV were administered six hours after the first batch, as the 20-minute whole blood clotting time (WBCT) remained positive. She was transfused with two units of fresh frozen plasma because of a deranged coagulation profile. Twelve hours following hospitalization, the patient’s vision improved, and the 20-minute whole blood clotting time normalized. Repeat ophthalmologic evaluation was normal, suggesting the cause of blindness to multiple occipital infarcts. But the patient remained anuric, and hemodialysis was initiated on the second day of hospitalization in view of an acute kidney injury.

Serial investigations revealed anemia, thrombocytopenia, and elevated renal parameters, although a decremental trend was noted in coagulation parameters, which eventually normalized. The hematologic evaluation was suggestive of microangiopathic hemolytic anemia with a hemoglobin nadir of 8 g (elevated lactate dehydrogenase, schistocytes in peripheral smear) and thrombocytopenia with a platelet nadir of 80,000 cells/cu mm on the fourth day of hospitalization, which gradually improved. The patient was subjected to 12 sessions of hemodialysis in total during hospitalization. As per the clinical and pathological evidence, the patient met the thrombotic microangiopathy criteria (microangiopathic hemolytic anemia, thrombocytopenia, and acute kidney injury) secondary to snake envenomation, which is often overlooked and rarely reported. The patient was treated with ASV, hemodialysis, antimicrobials for limb cellulitis, and other supportive measures and was discharged home after a fortnight stay in the hospital with the advice to follow up with a nephrologist. The patient had normal vision, and hematologic investigations were within normal limits during discharge. She required biweekly dialysis following discharge, and her renal function normalized four weeks post-discharge. 

## Discussion

Cerebrovascular manifestations following snake envenomation are uncommon, and cortical blindness is rarely reported [3]. Possible etiologies for blindness following snake envenomation reported in the literature are snake venom ophthalmoplegia, acute angle closure glaucoma following capillary leak syndrome, vitreous hemorrhage, retinal hemorrhage, optic neuritis, post-neuritic optic atrophy, and occipital infarcts [5-7]. Our patient developed cortical blindness due to multiple bilateral infarcts. Ischemic infarcts can occur following snake envenomation due to disseminated intravascular coagulation, toxic vasculitis, and thrombotic microangiopathy, apart from hypercoagulable factors in snake venom [3]. Snake venoms are complex, heterogeneous poisons with multiple effects. Hemotoxicity and bleeding are important contributors to morbidity and mortality, and they can be broadly classified as procoagulants or anticoagulants in their mechanism of action. Basic pro-coagulation metalloprotease [8], procoagulant factors V and X, protease, and phospholipase A2 [9] in Russell’s viper venom have been demonstrated to have procoagulant effects. Russell’s viper bite-associated thrombosis in various organs, such as the heart and lungs, has been reported in the literature [10]. Apart from the threat of devastating coagulopathy, hemotoxic snake envenomation can also deceptively present with ischemic symptoms. One should consider the possibility of ischemic stroke in patients presenting with unusual manifestations following snake envenomation [11]. The improvement of cortical blindness on the subsequent day with complete restoration of vision is rarely reported. The possible reasons are attributed to neuroplasticity in the remaining cortical areas, which may partly compensate for the impaired visual functions [12].

The typical procoagulant coagulopathy of snake bites is venom-induced consumption coagulopathy (VICC). The major complication of VICC is bleeding, which can be fatal. In a subset of patients with VICC, thrombotic microangiopathy (TMA) features may develop [13]. Thrombotic microangiopathy is often overlooked in snake envenomation, erroneously attributing microangiopathic hemolytic anemia, acute kidney injury, and thrombocytopenia to disseminated intravascular coagulation [4]. Our patient presented with VICC, as evidenced by an elevated coagulation profile, a positive 20-min whole blood clotting time, elevated D-dimer, and hypofibrinogenemia within a few hours of envenomation. Venom-induced consumption coagulopathy improved following anti-snake-venin administration, but microangiopathic hemolytic anemia (schistocytes in the peripheral smear, elevated lactate dehydrogenase) and thrombocytopenia were noted on serial hematological evaluation, and renal failure persisted, requiring multiple sessions of renal replacement therapy for anuria. The patient fulfilled the criteria for snake bite-associated thrombotic microangiopathy, possibly acquired hemolytic uremic syndrome.

The exact mechanism of TMA following envenomation is unclear, but it has been proposed that a toxin in venom may initiate TMA by inducing endothelial damage. Snakebite-associated TMA has been described in a variety of snake species known to cause VICC across all inhabited continents of the world [14-15]. Snakebite-associated TMA appears to be specifically associated with acute kidney injury (AKI) [16-17], in contrast to the significant complication of VICC, i.e., bleeding. Most of the patients require a long duration of renal replacement therapy and a longer hospital stay [4]. Snake bite-associated TMA is self-limiting, and the role of therapeutic plasma exchange is unknown [13]. The risk of TMA should be considered in patients with snake bites who have acute kidney damage, thrombocytopenia, microangiopathic hemolytic anemia, and a normal coagulation profile. Early diagnosis, dialysis, and plasmapheresis in refractory cases can improve renal outcomes. Although most patients with snake bite-associated TMA survive and achieve dialysis-free survival, they are at risk of long-term chronic kidney disease. Long-term follow-up with a nephrologist is hence recommended [13].

## Conclusions

Ophthalmic manifestations following a snake envenomation are varied, and cerebrovascular infarcts as a cause of blindness should be strongly suspected in patients with unremarkable fundoscopy and cranial nerve examination findings. Antivenin and supportive measures remain the primary treatment modality for patients presenting with snake envenomation. Some patients with venom-induced consumptive coagulopathy may develop thrombotic microangiopathy, which often clinically manifests as microangiopathic hemolytic anemia, acute kidney injury, and thrombocytopenia. Snake bite-associated thrombotic microangiopathy, although a self-limiting illness, can have a protracted hospital course. A high index of suspicion and early initiation of dialysis and plasmapheresis in refractory cases may improve renal outcomes.

## References

[1] Mohapatra B, Warrell DA, Suraweera W (2011). Snakebite mortality in India: a nationally representative mortality survey. PLoS Negl Trop Dis.

[2] B R H, L H, A J L, P K C, K B V (2013). A study on the clinico-epidemiological profile and the outcome of snake bite victims in a tertiary care centre in southern India. J Clin Diagn Res.

[3] Abraham A K, John L (2019). Hemotoxic snakebite presenting with bilateral blindness due to ischemic occipital infarcts. Indian J Crit Care Med.

[4] Rao IR, Prabhu AR, Nagaraju SP, Rangaswamy D (2019). Thrombotic microangiopathy: an under-recognised cause of snake-bite-related acute kidney injury. Indian J Nephrol.

[5] Mustapha SK, Mubi BM, Askira BH (2010). Bilateral blindness following snakebite. Trop Doct.

[6] Menon V, Tandon R, Sharma T, Gupta A (1997). Optic neuritis following snake bite. Indian J Ophthalmol.

[7] Sahai AS, Sinha RH (1978). Bilateral blindness following snake bite. Indian J Ophthalmol.

[8] Mukherjee AK (2008). Characterization of a novel pro-coagulant metalloprotease (RVBCMP) possessing alpha-fibrinogenase and tissue haemorrhagic activity from venom of Daboia russelli russelli (Russell's viper): evidence of distinct coagulant and haemorrhagic sites in RVBCMP. Toxicon.

[9] Warrell DA (1989). Snake venoms in science and clinical medicine. 1. Russell's viper: biology, venom and treatment of bites. Trans R Soc Trop Med Hyg.

[10] Hung DZ, Wu ML, Deng JF, Yang DY, Lin-Shiau SY (2002). Multiple thrombotic occlusions of vessels after Russell's viper envenoming. Pharmacol Toxicol.

[11] Subasinghe CJ, Sarathchandra C, Kandeepan T, Kulatunga A (2014). Bilateral blindness following Russell's viper bite - a rare clinical presentation: a case report. J Med Case Rep.

[12] Shibuki K, Wakui I, Fujimura T, Tomikawa M, Hasegawa S (2020). Rapid recovery from cortical blindness caused by an old cerebral infarction. Front Neurol.

[13] Noutsos T, Currie BJ, Wijewickrama ES, Isbister GK (2022). Snakebite associated thrombotic microangiopathy and recommendations for clinical practice. Toxins (Basel).

[14] Herath N, Wazil A, Kularatne S (2012). Thrombotic microangiopathy and acute kidney injury in hump-nosed viper (Hypnale species) envenoming: a descriptive study in Sri Lanka. Toxicon.

[15] Dineshkumar T, Dhanapriya J, Sakthirajan R, Thirumalvalavan K, Kurien AA, Balasubramaniyan T, Gopalakrishnan N (2017). Thrombotic microangiopathy due to Viperidae bite: two case reports. Indian J Nephrol.

[16] Wijewickrama ES, Gooneratne LV, Gnanathasan A, Gawarammana I, Gunatilake M, Isbister GK (2021). Severe acute kidney injury following Sri Lankan Hypnale spp. envenoming is associated with thrombotic microangiopathy. Clin Toxicol (Phila).

[17] Isbister GK, Little M, Cull G (2007). Thrombotic microangiopathy from Australian brown snake (Pseudonaja) envenoming. Intern Med J.

